# Contracting private sector providers for public sector health services in Jalisco, Mexico: perspectives of system actors

**DOI:** 10.1186/1478-4491-7-79

**Published:** 2009-10-22

**Authors:** Gustavo H Nigenda, Luz María González

**Affiliations:** 1 Health Services and Systems Innovations, Health Systems Research Centre, National Institute of Public Health, Cuernavaca, Morelos, Mexico; 2 Management and Leadership, Health Systems Research Centre, National Institute of Public Health, Cuernavaca, Morelos, Mexico

## Abstract

**Introduction:**

Contracting out health services is a strategy that many health systems in the developing world are following, despite the lack of decisive evidence that this is the best way to improve quality, increase efficiency and expand coverage. A large body of literature has appeared in recent years focusing on the results of several contracting strategies, but very few papers have addressed aspects of the managerial process and how this can affect results.

**Case description:**

This paper describes and analyses the perceptions and opinions of managers and workers about the benefits and challenges of the contracting model that has been in place for almost 10 years in the State of Jalisco, Mexico.

Both qualitative and quantitative information was collected. An open-ended questionnaire was used to obtain information from a group of managers, while information provided by a self-selected group of workers was collected via a closed-ended questionnaire. The analysis contrasted the information obtained from each source.

**Discussion and Evaluation:**

Findings show that perceptions of managers and workers vary for most of the items studied. For managers the model has been a success, as it has allowed for expansion of coverage based on a cost-effective strategy, while for workers the model also possesses positive elements but fails to provide fair labour relationships, which negatively affects their performance.

**Conclusion:**

Perspectives of the two main groups of actors in Jalisco's contracting model are important in the design and adjustment of an adequate contracting model that includes managerial elements to give incentives to worker performance, a key element necessary to achieve the model's ultimate objectives. Lessons learnt from this study could be relevant for the experience of contracting models in other developing countries.

## Introduction

This article presents results from a research project that analyses the performance of a model implemented by the Ministry of Health (MOH) of the State of Jalisco, Mexico, for contracting private providers with public funds. This model is a strategy employed by the Jalisco MOH to extend coverage to populations without access to formal health services and to increase the efficient use of available resources. With the information gathered through a study that carried out a comprehensive analysis of the model, this document presents the perspectives of service providers and decision-makers regarding the model's capacity to meet objectives, their particular form of participation in the model and issues related to the managerial process of contracting.

An increasing number of studies in the health literature document health services contracting by government authorities [1]. These studies report diverse strategies to link the public model and the private actors, as well as the various consequences (positive and negative) in terms of coverage, efficiency, equity and quality of care. These models have been favored particularly by developing countries and some have been evaluated [2-6]. An important number of these models have been promoted and even financed by international development agencies. Yet few have mobilized national or local fiscal resources for implementation; one notable exception is Costa Rica [7].

Furthermore, few articles focus on the managerial aspects of contracting-out [8]. Management can be regarded as a process to carry out allocative, costing, standardizing and purchasing decisions and activities to achieve institutional goals, but it also includes a whole set of aspects related to labour relationships, most of which are key to the attainment of goals.

Underlying the attainment of efficiency and quality of care, the way in which workers are linked to the model is crucial to guarantee adequate performance. Addressing workers takes into consideration not only juridical and economic aspects but also motivational elements to harmonize relationships between management and workforce. These issues belong to what some authors understand as "organizational culture" [9].

For example, in the Jalisco MOH, putting innovations into practice is an expression of its organizational culture. The MOH of Jalisco is known nationwide for introducing changes - within the limits that the law imposes on public institutions - to improve the provision of services and respond to population needs. The present paper focuses on the latter aspects and aims to depict and analyse a set of opinions and perspectives provided by two main groups of actors, managers and workers, about a managerial innovation represented by the contracting-out strategy. The analysis of these opinions will allow us to identify differential views on similar issues, and identify challenges and opportunities that can lead us to improve the managerial process of contracting-out.

Within this scope, the evidence provided by the study results can potentially benefit Mexico and many other developing countries embarking on the process of contracting-out health services. The study highlights a group of risks that can impede the attainment of the contracting model objectives. Among them are included the decay in labour conditions, the lack of incentives for contracted personnel and the administrative workload represented by the surveillance of productivity to estimate payment levels. Based on the previous statement, some of the main lessons highlight the need to create a system of incentives to promote a balance between efficiency and quality, and to implement a clearly defined monitoring system operated by adequately trained personnel to carry out this complex task.

### The reform of the Mexican health care system and the onset of contracting-out

Since 1943 the Mexican health care system had adopted a segmented structure separating social security from the public system [10]. By the 1980s the system entered into a deep crisis expressed by its inability to fully cover the population and by the under-financing of the public system.

As a response to the crisis, the system initiated an important stage of reform. Various authors agree that the decentralization of health services in the early 1980s - the first stage of the reform - resulted in a chain of dynamic changes still felt today. These changes sought to address the needs of the population group not covered by social security services [11].

Decentralization was an important and strategic phase to redistribute the financial responsibility for health care between the states and the federal level. The process was initiated in states with greater local, financial, human and material resources, but it encountered difficulties and was interrupted from 1988 to 1994. In 1995, decentralization was reinitiated in a second stage and was declared completed in 1999.

Although decentralization itself did not resolve the financial and equity-related problems of the health system [12], over time some states have taken advantage of the process to increase their decision-making capacity regarding resource allocation, thus reducing the role of the federal government in this sphere.

Furthermore, this increase in autonomy promoted innovation that was primarily financed with state funds. State health bureaucrats, or technocrats, began to play a role in the conceptualization of the local system [3]. These technocracies have been structured around state governments; it is not possible to distinguish which political party most supports them [13].

In the case of the State of Jalisco, located in western Mexico, this group of health technocrats comprised individuals with solid academic training at Mexican and international institutions, and with significant experience in the political sphere. Main sociodemographic and health features of Jalisco appear in Table 1. Jalisco was decentralized during the second period; however from 1983 to 1995, prior to its decentralization, different projects had been put forth. In many cases these projects (e.g. the Mental Health Model) became points of reference for other states.

**Table 1:** Characteristics of the State of Jalisco (circa 2005)

	**Mexico**	**Jalisco**
GNP per capita (US dollars)	7,143.95 (2006)*	6,797.26 (2006)*

% population covered by social security	47,193,861 (45.6% of Mexican population) (2006)**	3,516,645 (51.3% of Jalisco population) (2006)**

Physicians per 1000 inhabitants	1.3 (2004)	1.3 (2004)

Health expenditure as % of GNP	2.9 (2006)***	3.1 (2006)***

Population	104,874,282 (2006)*	6,843,469 (2006)*

*Source:  [Consulted 27/10/2009]

** Source:  [Consulted 27/10/2009]

*** Source: Salud México 2001-2005. Statistical Annex 1.12 (p. 182)

Early on, Jalisco started to investigate the option of incorporating private sector participation in the public health system. The state is characterized by a high level of industrial development, influenced by free-market thinking in the economic and political spheres. In this context the idea of total state dominion over public policy is less acceptable than in other states [14]. As such, the possibility of private participation in the public health sector did not meet with resistance by the stakeholders, as in other states. Currently this strategy has been incorporated in different MOH programmes in Jalisco.

### History and general characteristics of the model

In the mid-1990s, technocrats in the Ministry of Health of the State of Jalisco had realized that despite efforts to extend health care coverage through federal programmes, there were still population groups, particularly in rural and semi-urban areas that did not have continuous access to primary and secondary level health care. There were two options to extend coverage: (1) construct new units in the public health network, or (2) contract private providers. The latter was favoured after costing exercises showed that building additional infrastructure was not financially viable.

To carry out the decentralization process in Jalisco, the state and federal authorities agreed to create an institution called the Decentralized Public Agency (DPA), which could carry out functions that the Secretary of Health was forbidden by law to carry out, such as the contracting of private sector services and providers. In practice the DPA and the Jalisco MOH coordinated with each other in order to carry out the duties of the health sector and generally the same person headed both organizations.

In 1997, the state government earmarked budgetary funds that would finance and permit the operation of the new programme to contract health teams and hospitals to provide services to the population not covered by a social security institution. As a requisite for contracting, two types of services were selected: a basic health unit that consisted of a physician, nurse and health technician who worked as a team to provide health services in rural areas. General hospitals that offered basic specialties (surgery, paediatrics, gynaecology/obstetrics and internal medicine) made up the second provider type.

The basic health unit contracted personnel for a defined period of time (usually three months) through renewable contracts. Payment varies by job category. For example, physicians receive a fixed salary equivalent to 50% of the permanent MOH physician salary. The remainder is variable and calculated based on monthly productivity (defined primarily as the number of consultations). The other categories of health personnel also receive a fixed salary complemented by productivity payments, based on indicators related to their activities (e.g. number of home visits, immunizations administered).

By 2002, MOH bureaucrats looked for a budgetary increase to incorporate more basic units and hospitals into the contracting model. In negotiations with the State of Jalisco Treasury, the source of all resources for the model, MOH top managers put forth three main arguments: (1) the contracting model was able to expand coverage to communities without health care access, (2) it was cheaper to contract-out providers than build units and hire permanent personnel, and (3) in order to boost the positive effects of the model, a financial increase was needed. These arguments were founded both politically and technically and finally the decision was made to expand the model's coverage. The budget increased by nearly 100% between 2002 and 2004; the majority of this additional investment went to expanding the number of basic health units.

A key technical aspect of the model is the regulatory mechanism. This mechanism is complex: one of its goals is to calculate the precise amount of the additional productivity-based payment made to health personnel. Each month the basic health unit personnel report their productivity to the health jurisdiction, which then forwards the reports to the central coordinating offices in Guadalajara. Based on productivity reports, the coordinating office estimates the additional payment. Statistical records are maintained at the central level to monitor performance over time. When a provider surpasses the monthly average, a technical audit of the basic health unit or hospital goes into effect. This purpose of the audit is to understand the change in performance and is based on a review of patient charts maintained by health personnel. A systematic monthly audit is also carried out in randomly selected units in order to review productivity and medical charts.

The basic health units are distributed throughout Jalisco, primarily in localities that lack a public health centre. The specific criteria are that these units be located in localities with a population of no more than 2500, without local public health services and with the nearest public health clinic more than one hour away via public transportation.

Undoubtedly, the topic of private sector incorporation in the development and implementation of public health policy has been widely debated; its consequences have not always been positive. The data presented in this article focus on the perspectives of the primary care providers and other key actors involved in the managerial process of the contracting-out model, considering the model's advantages and disadvantages to provide services to populations in rural areas where public units were not available.

## Case description

### Data collection

In 2004, the Mexican Health Foundation initiated a study of the diverse models of public-private interaction in Mexico's health sector. The case of Jalisco proves interesting because there are currently few models of public-private interaction for primary care service provision in the country. A case study was carried out with the aim of describing the model's legal framework, financial mechanisms, linking of private health care providers in the public network and participation by health personnel.

To develop the case study, a set of qualitative, quantitative and documenting techniques was applied with the aim of gathering data to describe the model's origin, legal framework, financial mechanisms and contracting of private health providers. The participation and perspectives of health personnel involved in the provision of services were also documented, along with the use of contracts as regulatory mechanisms, the supervision and control systems, user satisfaction with health care and the general model outcomes. Through a descriptive analysis and triangulation of the information obtained from different sources, researchers were able gain in-depth knowledge about the model's operation as well as the perspectives of the actors involved.

Fieldwork was carried out between 2004 and 2005. The population under study consisted of two main model participants: decision-makers (at the Jalisco Ministry of Health), private providers (doctors, nurses and health promoters as well as MOH hospital managers).

To document the perspectives of private providers, a questionnaire was administered to a self-selected group comprising doctors, nurses and health technicians. The questionnaire included the following topics: (1) sociodemographic profile, (2) motivations for accepting the contract, (3) working conditions, (4) opinions about supervision and indicators employed, and (5) opinion of user satisfaction at their health unit. The instrument also collected data on contract workers' future expectations about their labour conditions and opinions about the strengths and weaknesses of the contracting model.

The informants were selected in three stages. First the universe was defined considering 180 contracted workers in all three categories (physicians, nurses and health promoters), as documented in Jalisco MOH records. Second, the questionnaire was mailed to all contracted health professionals. Third, the completed questionnaires were returned within one week to the payment office of the corresponding health jurisdiction. Questionnaires were then delivered to the model's managers in Guadalajara and finally to the researchers. From the total of 180 questionnaires, only 87 were completed and used for analysis. Self-selection of the group did not allow researchers to make any kind of inferential analysis.

Although the way in which the respondent group was constructed is a major limitation for the interpretation of the results, the group showed homogeneous characteristics that responded to the criteria previously established by researchers, namely: (1) all personnel were included in the list of contracted personnel provided by the SSJ, (2) all had the same contractual and payment conditions, (3) all were supervised under the same scheme and (4) all were located in rural and semi-urban areas without a public health unit.

For the qualitative component, a total of 29 interviews were carried out: seven interviews with top managers at the Jalisco MOH, four with owners/managers of the hospitals under contract and 18 with users of health services units staffed by contracted private providers. Interviews were semistructured. They lasted 60 minutes on average and were conducted and audio-recorded by a team of three field researchers. Informed consent for all informants was obtained prior to the beginning of the interview. The selection of informants in the qualitative component was purposeful and intentional, aiming to obtain the most relevant information possible for the objectives of the research project. Participants were selected according to the degree to which they met the criteria originally defined by the project. A common criterion for inclusion for all informants was that they should possess knowledge of and have direct participation in the model. Data were processed by means of Atlas Ti software.

The information obtained from the different methodologies was contrasted and triangulated in order to confirm the results and the analysis of the case study.

### Health personnel profile

The numbers of basic health units and contracted personnel have increased in recent years. In 2003, 40 physicians were contracted to work in the basic health units. By 2004 this number had increased to 180 persons, including physicians, nurses and health promoters. This group is organized into 52 basic health units working in 12 health jurisdictions. Contracted physicians are generally young residents, recent graduates or people who work as substitutes, filling in for those on maternity leave, vacation or some other type of leave. The nursing and health promoter staff usually live in the communities they serve, are known by the community, and in some cases have previously worked for the state MOH in a related area.

In reviewing the profile of the 87 contracted workers who completed the survey, two points stand out: the majority of contracted personnel are female (80%) and the distribution of completed surveys among categories of health personnel are relatively homogeneous (physicians 34.5%, nurses 31% and health promoters 34.5%). As detailed in Table 2, compared to the overall number of contracted personnel, self-selection was consistent between occupational groups, but not by sex. However, the differences by sex in completion of the survey are observed exclusively in the health promoter category. Additionally, more than half of all personnel in all categories (55%) have earned a university degree. The contracted staff ages range from 21 to 50 years old, with an average age of 30.

**Table 2:** Differences between total personnel contracted in basic health units and the self-selected group of respondents

**Occupational category**	**Total**	**%**	**M**	**F**	**Sample**	**%**	**M**	**F**
Physicians	71	39	32%	68%	30	34	30%	70%

Nurses	49	27	1%	99%	27	31	4%	96%

Promoters	60	33	55%	45%	30	34	17%	83%

Total	180	100			87	100		

Source: Authors, with data from the health services providers' questionnaire. Research project on public-private interactions in the Mexican health sector.

Also worth noting is that 82% of the self-selected group indicated work experience prior to joining the basic health units. Of this group, 54% had worked in the public sector and the other half in the private sector. Likewise, 45% of those who had previous work experience worked, on average, between one and two years in their previous job.

The information presented in the following section emerges from a mix of primary and secondary sources. All tables and figures were produced from information collected by the survey of practitioners and completed with information obtained from MOH records.

### Opinion of contracted personnel about the advantages and disadvantages of participating in the model

The basic health units require physical workspace for service provision, equipped with adequate instruments and supplies. The MOH negotiated with municipal authorities, reaching an agreement whereby the municipalities would supply the physical workspace and the Secretary would supply the equipment and medications through the public health supply system.

**Figure 1:**
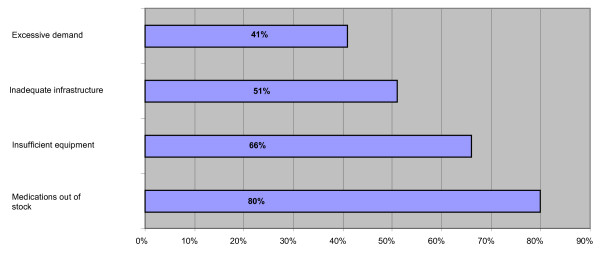
**Main service delivery problems faced by basic health unit personnel**. Source: Authors, with data from the health services providers' questionnaire. Research project on public-private interactions in the Mexican health sector.

The result is working conditions that are not always adequate, thereby reducing the capacity of the basic health units to provide care in this minimal setting. According to Figure 1, supply of medications is the biggest problem encountered by health personnel, highlighted by 80% of the respondents. Lack of equipment and inadequate physical space in terms of size, design, ventilation and lighting were also important problems. Excess demand was not mentioned as a major problem, given that more demand generates greater income based on productivity.

Regarding the contractual agreements between the institutions and private providers, two important aspects stand out: the extent to which the personnel considered advantageous (or disadvantageous) the contracting mechanism, and the way in which salaries are calculated. Sixty-one percent of the health workers considered the contracting process somewhat advantageous, 29% considered it disadvantageous or very disadvantageous and only 7.6% considered the mechanism advantageous or very advantageous. This distribution suggests that the contracting process is viewed ambiguously; health workers perceive both the positive (flexibility in time) and negative aspects (income level).

Table 3 shows the reasons the personnel qualified the contract mechanism as advantageous or disadvantageous. The questionnaires provided the opportunity for respondents to spontaneously mention the reason underlying the qualification. The majority of the reasons for a negative opinion of the contracting process are related to personnel issues such as salary, lack of benefits, the duration of the contract and the impossibility of obtaining a long-term position. None of the reasons given for a disadvantageous ranking mentioned the inability to provide quality care, geographical proximity to the population receiving care or the supervision that they receive. The qualification of "somewhat advantageous" also centres on personal reasons, but includes other reasons, such as combining this job with other activities or simply having the opportunity to work. The "advantageous" category is the only one that considers geographical proximity to the target population. In Table 3, each of the reasons within the three categories is ranked according to the level of priority (1 to 10) that each informant defined. The average priority level estimated for all informants was used to rank them.

**Table 3:** Basic health unit personnel reasons for characterizing the contracting mechanism as advantageous or disadvantageous

**Category**	**Reasons**
Somewhat advantageous	1. Salary and benefits drawbacks
	2. Cannot accumulate seniority
	3. Renewing contracts is dependent on productivity
	4. Untimely salary payments
	5. Short contract period (three months)
	6. Job insecurity
	7. Few benefits
	8. No medical insurance
	9. No refresher or continuing education courses
	10. Greater workload than those with permanent position
	11. Can combine this job with other activities
	12. Opportunity to work

Disadvantageous or very disadvantageous	1. Fewer rights than permanent personnel
	2. Cannot accumulate seniority
	3. Short contract period
	4. Salary and benefits drawback
	5. Greater workload than those with permanent position
	6. Job insecurity
	7. Undefined job activities
	8. No social security benefits
	9. Untimely salary payments

Advantageous or very advantageous	1. Productivity payments
	2. Recent provision of health insurance
	3. Ability to work in their community
	4. Opportunity to work

Source: Authors, with data from the health services providers' questionnaire. Research project on public-private interactions in the Mexican health sector.

An underlying theme among the range of the opinions is that those who accept contracts by the MOH do so with the short- or medium-term goal of obtaining a permanent position, with the accompanying benefits and rights that the unionized, permanent workers enjoy. One non-explicit factor related to aiming for a permanent position - in addition to those points already mentioned in Table 3 is resistance to having payments and incentives based on productivity and quality standards. In Table 4 the preference of contracted personnel for a permanent position is clear, a finding that is valid across the three health personnel categories. However, an important group of contractors prefers to maintain their current status for an indefinite period. The proportion of groups expressing other preferences is marginal. Finally, worth highlighting is that among physicians, the possibility of working independently in a private doctor's office is mentioned by only a small proportion of cases.

**Table 4:** Basic health unit personnel preferences regarding contracts

**Preference of health unit personnel regarding contracts**	**% Basic health unit personnel**			
	
	**Total**	**Physicians**	**Nurses**	**Others**
Maintain indefinitely	17	21	22	13

Maintain until finding other job	1		4	

Obtain a permanent position with the State Health Secretary	67	70	55	77

Obtain a permanent position in another public institution	2		4	3

Work independently, in profession	1	3		

Other	2	3	4	

Don't know; no response	10	3	11	7

Total	100	100	100	100

Source: Authors, with data from the health services providers' questionnaire. Research project on public-private interactions in the Mexican health sector.

**Figure 2:**
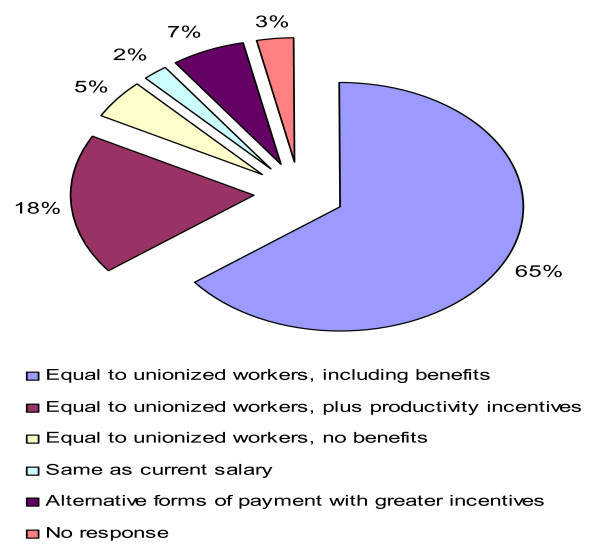
**Basic health unit personnel payment preferences**. Source: Authors, with data from the health services providers' questionnaire. Research project on public-private interactions in the Mexican health sector.

Salary preferences reflect in part the previous tendency. The majority of personnel would prefer a salary that is constructed differently. As mentioned, personnel payments comprise a base salary (50% of total possible earnings), to which productivity payments are added. This model allows managers to promote productivity and efficiency. Among permanent workers, productivity is measured but is not used for sanctions, in the case of low productivity, or bonuses for high productivity. Incentives are paid to salaried workers based on punctuality, which is not a factor affecting efficiency. According to Figure 2 a large group of contracted personnel (65%) suggests that they should earn the same salary as permanent workers, excluding productivity as a factor for calculating income. Another important group (18%) would prefer the same salary as permanent workers, without taking into account benefits. The remaining groups represent small percentages; there is a small group that considers their income level to be fair.

### Managers' perspective

The opinions of the health care providers - physicians, nurses and promoters - are fundamental for understanding the achievements and difficulties in operating the model. However, their vision centres on the advantages and problems related to their participation and underestimates the implications for other actors. Thus the fieldwork carried out for this study also gathered information from managers.

As stated previously, the two most important objectives of this model are to widen coverage to underserved areas lacking public health infrastructure and to employ resources using strict efficiency-based criteria. In order to meet the first objective, the specific areas for deployment of basic health units must be defined, which to date has largely been achieved. It is the state MOH, and not the contracted personnel, that defines the locations for the teams. The strategy to accomplish the second objective is through ensuring a competitive salary, similar to that of unionized workers, initially without considering other benefits such as social security, pensions, etc. However, health authorities have pointed out an important increase in the workload of medical personnel and managers assigned to measure the productivity and supervise personnel under contract. The lack of trained personnel to carry out this task could represent an obstacle to its sustainability.

According to state MOH authorities, achieving the model's objectives is more important than the way it functions. They consider the contracting of health personnel both fundamental and instrumental to meeting objectives. State as well as jurisdictional authorities consider that basic health unit productivity can be 100% greater than that of the public health network. They cite, for example, that the average number of consultations per day in a basic health unit is between 20 and 30, and that in a standard public health centre it ranges from 10 to 14.

Other aspects highlighted by these informants are related to a strong commitment of contracted personnel to their job: combining a sense of responsibility and respect for supervisors, meeting immunization goals (a productivity measure), increasing service demand, greater prenatal care coverage resulting in a decreased risk of maternal mortality, and increased efficiency in resource allocation. Managers also mentioned the high level of satisfaction among the health units' target population, this being one of the model's corollary achievements. Indeed, for many sites the services provided by these units constitutes the first time that formal health care services have been offered in a continuous manner in their area.

Managers appreciate that they have a high level of control over health unit performance through the use of indicators concerning personnel activities. An important issue with the contracting model is that the union, a powerful actor in the negotiation of labour conditions for workers, has been limited to recommending personnel to be contracted. The final decision as to how contracting is carried out rests with the state health authorities.

## Discussion and Evaluation

This paper describes the perception of managers and contracted workers participating in the operation of a model that contracts private providers using public funds in the State of Jalisco, Mexico. This contractual model is unique in Mexico, yet it has been inspired by similar initiatives in other developed and developing countries [15]. During its 10 years of existence, the model has advanced in various aspects. It has mobilized significant public resources from the state-level Treasury, permitting a greater number of basic health unit personnel to be contracted. As a consequence, coverage of populations living in rural areas has increased.

It must be stated initially that contracting has a quite specific connotation in the Mexican health system. For most managers and workers, to contract a person means to establish a labour relationship that makes that person an employee of the institution. For most contracted workers, this way of engaging the institution is the first step to becoming a permanent worker, although this may not necessarily be the case. Contracting has a distinctively different meaning in other contexts.

In England, for example, where general practitioners are contracted to provide primary care services, they regard themselves as private contractors who have the freedom to maintain an engagement with the public sector or to run their private business. Despite changes that occurred in the 1990s regarding the organization of group practices and the system of incentives and clinical performance, the basic idea of the general practitioner (GP) as a contractor and not as a salaried worker remains [16]. This difference permeates the understanding of the contracting-out model.

A further difference that should be highlighted is that English GPs are paid under a capitation scheme to provide incentives for promotion and prevention activities. No contracting-out scheme in Mexico has attempted to pay under a capitation scheme and the Jalisco MOH is no exception. Workers in this model are paid a basic allowance and on top of this an extra payment for productivity. Liu X [17] show that most contracting-out schemes do not consider capitation as the main option for paying providers. Setting up a capitation payment scheme is neither administratively nor culturally appealing in the Mexican context.

As shown in other studies [18], the perception of any given phenomenon varies according to the position that each actor has within the institution. The organizational culture that prevails in the Jalisco MOH includes the possibility of developing innovations to improve the performance of the health system. Innovations are generally proposed by managers but not always accepted by all actors involved, and the study shows that there are many issues involved in implementing the model that are not valued equally by actors in different positions in the institutional structure. The results presented above confirm the variation in the perceptions of different types of actors.

The model's managers focus on the achievement of the model's objectives, highlighting results in the form of increased coverage of populations who, prior to the model's implementation, had no access to formal health services, as well as the efficient use of resources based on the differences in the productivity/investment ratio between contracted and public units. Labour conditions of contracted personnel and the effect of these conditions on their productivity and quality of care are not relevant issues in their discourse.

Unlike managers, contracted personnel focus on the conditions under which workers are contracted. Even though contracted personnel do not outright reject or critique the model, the desire to obtain better working conditions and job security is clear. Workers clearly seek a permanent staff position in the Ministry of Health. From their perspective, the increase in coverage and efficient use of resources does not represent a great achievement of the model.

This gap in actors' perspectives has important medium- and long-term implications for the model. Extending coverage is an unquestionable achievement, but the achievement of efficiency less so. The positive relation obtained between investment and productivity diminishes the possibility of increasing the investment to improve workers' labour conditions. Increasing economic incentives, medical care, bonus payments and even ensuring continuity and stability in the contracted position require greater financial investment.

Given the nature of the work, it is important to provide an appropriate job offer and package of benefits. In this sense, contracting models should consider provision of these benefits to be a productive investment. However, managers should maintain the prerogative to monitor and supervise the performance of contracted workers in order to ensure high quality of care over time.

According to Dal Poz, by the year 2000 in Brazil there were different modalities of contracting health workers, all with advantages and disadvantages for managers and workers. Most of these contracts provided flexible working conditions for employees, yet normally did not meet the country's legal labour requirements. This trend demonstrates the advancement of structural reforms and the impact they are having on the labour conditions of health workers [19]. Preserving adequate labour conditions is fundamental when contracting of health workers is undertaken.

The perspective of managers and workers regarding contracting-out services has not been widely documented internationally. In Canada, it was found that the contracting of more than 8000 workers in British Columbia since 2002 has produced more negative effects than positive, according to the workers' perspective [20]. Among those issues relevant to workers are low pay, meager benefits, heavy workloads, poor training and no job security.

A report published by USAID/PSP-One [21] points out the importance of an adequate managerial strategy in the contracting-out process for reproductive health services. Drawing lessons from Bangladesh, Cambodia and Guatemala, the document stresses the relevance of transforming the ideological position of managers in order to make them capable of undertaking their new functions as contractors of services, including the negotiation of contracts and the monitoring of contractor performance. The document also points out the necessity of good coordination between purchasers and providers in order to prevent conflicts. The parties, according to the document, should mutually develop performance goals, identify potential sources of conflict and establish cooperative ways to resolve problems that may arise during contract performance. Communication is part of a successful managerial strategy.

## Conclusion

As these results are generated from an exploratory study, the findings are not conclusive. However, findings clearly point out the importance of acknowledging the goal achievement perspective expressed by managers and the labour rights perspective expressed by workers. No doubt both are necessary and reconcilable. Achieving efficiency should not be an objective to attain at the expense of making vulnerable the rights of workers to have a decent income and benefits under the given labour regulations of countries.

It is likely that the model has had an effect on productivity, quality of care and efficiency in the provision of services. The performance of the state MOH has been important from both the technical and political perspectives. Technically, there are three key aspects in the model's operation. The first is the contracting mechanism, which allows the state MOH to determine the geographical location of the basic health units and to ensure their permanency, traditionally the Achilles' heel of the country's primary health care system. Health personnel are usually reluctant to relocate to far-removed communities and be subjected to productivity measures. The second key aspect of the model's success is the ability to link productivity to salary payments, thereby increasing the number of services offered and optimizing resources. The third element, and perhaps the most important for the achievement of efficiency, is the implementation of a strict regulatory and oversight system, which punctually and systematically reports personnel productivity, thereby permitting negotiation and discussion of those instances where productivity falls outside the norm.

Politically, the health authorities have been able to implement a model for almost 10 years with the support of the state Treasury authorities, and have benefited from a budgetary increase in recent years. They have also established an agreement with the health workers' union, obtaining the union's tolerance of the model.

Labour rights for contracted workers and the model's rationale for widening health care coverage and increasing quality and efficiency of resource allocation are not irreconcilable. In fact, the model has shown flexibility through introducing modifications that allow workers to increase their benefits. Recently the state Ministry of Health and the contracted workers have been negotiating benefits such as the provision of a major medical health insurance plan and the possibility of making payments into a personal retirement fund. Doubtless, these improvements in working conditions could produce a positive impact on fundamental aspects such as the long-term sustainability of the model, political support of workers for the model, the development of a quality-of-care culture in which worker satisfaction plays an important role and the possibility of replicating the model in other regions in Mexico and other developing countries in a consistent and viable manner to extend health services to the poor.

The results presented and discussed in the paper may be relevant for other experiences in developing countries. Thousands of workers are being contracted on a temporary basis today. Health systems rationalists trust that contracting could be a good option to improve health services performance. However, the decision to contract-out health services should follow a cautious approach in which the opinions of directly involved actors are considered in the implementation strategy.

## Competing interests

The authors declare that they have no competing interests.

## Authors' contributions

GN designed the overall study and the data collection instruments, analysed information and participated in the drafting of the document. LG participated in the design of data collection instruments, collected information in the field, systematized information and participated in the analysis and drafting of the document. Both authors read and approved the final manuscript.

## Authors' information

GN is Director of Health Services and Systems Innovations, Health Systems Research Centre, National Institute of Public Health. LG is a Researcher, Health Systems Research Centre, National Institute of Public Health.
